# Interpretive Diversity Understanding, Parental Practices, and Contextual Factors Involved in Primary School-age Children’s Cheating and Lying Behavior

**DOI:** 10.3390/ejihpe12110114

**Published:** 2022-11-11

**Authors:** Narcisa Prodan, Melania Moldovan, Simina Alexandra Cacuci, Laura Visu-Petra

**Affiliations:** Research in Individual Differences and Legal Psychology (RIDDLE) Lab, Department of Psychology, Babeș-Bolyai University, 400015 Cluj-Napoca, Romania

**Keywords:** cheating, lie-telling behavior, interpretive diversity understanding, parental rearing practices, bilingualism, SES, school

## Abstract

Dishonesty is an interpersonal process that relies on sophisticated socio-cognitive mechanisms embedded in a complex network of individual and contextual factors. The present study examined parental rearing practices, bilingualism, socioeconomic status, and children’s interpretive diversity understanding (i.e., the ability to understand the constructive nature of the human mind) in relation to their cheating and lie-telling behavior. 196 school-age children (9–11 years old) participated in a novel trivia game-like temptation resistance paradigm to elicit dishonesty and to verify their interpretive diversity understanding. Results revealed that children’s decision to cheat and lie was positively associated with their understanding of the constructive nature of the human mind and with parental rejection. Children with rejective parents were more likely to lie compared to their counterparts. This may suggest that understanding social interactions and the relationship with caregivers can impact children’s cheating behavior and the extent to which they are willing to deceive about it. Understanding the constructive nature of the mind was also a positive predictor of children’s ability to maintain their lies. Finally, being bilingual and having a higher socioeconomic status positively predicted children’s deception, these intriguing results warranting further research into the complex network of deception influences.

## 1. Introduction

A fundamental premise of children’s social development is the ability to achieve various self-directed goals while adhering to social norms. Following social rules and expectations represents one of the most important social behavior children learn early at home, and later in school [1]. However, despite constant encouragement to follow them, children still have difficulties negotiating between their early egocentric tendencies and social requirements with implications in various settings, such as school environment. For instance, previous research has documented high rates of cheating and lying about it from an early age when children primarily seek to avoid imminent punishment or to obtain a personal gain [2]. Dishonesty is a normative part of a child’s development, being considered a marker of their cognitive competence [3,4]. In laboratory settings, children’s deceptive behavior has been studied using the seminal temptation resistance paradigm (TRP) [5], implemented either via Guessing games (frequently used in preschoolers) [6] or Trivia games (more suitable for older children) [7]. In the latter ones, participants are asked to respond to several multiple-choice questions in order to win a desirable prize. However, during the task, they are offered the possibility to cheat by peeking at the answers for the more difficult questions during a brief experimenter’s absence. 

The TRP paradigm addresses three deceptive acts that differ in their complexity. At first, children have to decide if they are going to peek or not at the correct answers, which involves *cheating*. Past research showed that children’s decision to cheat is highly related to their motivation to win, inhibitory control, or personality traits [8,9]. The decision to cheat sometimes comes with a second challenge – *lie-telling*. When deciding to lie or not about their transgression, school-age children are beginning to guide their decisions based on a quasi-rational process involving the plausibility principle. They are becoming increasingly capable of contrasting costs and benefits and deciding if it is worth taking the risk based on social context [10]. As such, even if children cheat on a game, they could decide not to lie about doing so if there is a chance to be easily discovered. Lastly, if lying occurs, children must be able to sustain that lie if the recipient decides to ask for details, generating what is known as *semantic leakage control* [11,12] which refers to one’s ability to maintain a good consistency between initial and subsequent statements to be credible [2,7,13]. Children’s ability to maintain their lies is not always guaranteed, younger children having difficulties maintaining their initial denials if questioned [13]. The differences between these three levels of dishonesty rely on different cognitive sophistication [14,15] and the motivation behind them. For instance, when cheating, children are mainly seeking to break a rule to gain an advantage; instead, when choosing to lie, they are trying to manipulate the other’s behavior or beliefs to escape punishment [16].

Regardless of the robust research examining the development of dishonest behaviors and their cognitive underpinnings, there is less work examining how social and contextual factors can contribute to dishonesty rates throughout childhood [3,4]. Dishonesty represents an interpersonal exercise shaped by socio-environmental factors as well as cognitive ones [17]. Past research indicated that while the cognitive factors associated with children’s dishonesty can shed light on *how* they succeed in deceiving others, social and contextual factors might tap into *when* children decide to act dishonestly or not [18]. In the current study we focused on investigating both cognitive (e.g., advanced theory of mind), social (e.g., parental practices), and contextual (e.g., bilingualism and socioeconomic status) factors that can shed some light on the mechanisms behind cheating, lie-telling, and semantic leakage control in school-age children.

Understanding the developmental origins of deception could shed light on the nature of children’s moral decision-making, informing interventions aimed at preventing the development of pervasive deceptive practices later on. By examining socio-cognitive and contextual factors associated with children’s cheating, lie-telling, and semantic leakage control, we indirectly contribute to the design of honesty-promoting interventions focused on the “deep structure” of deception [19]. We address the process behind deception, the social figures that can promote honesty, and the contextual factors that can contribute to this reinforcement of honesty in children. 

### 1.1. Children’s Dishonesty and Cognitive Factors: Theory of Mind (ToM)

Using various versions of the TRP task, studies yielded mixed results regarding ToM’s involvement in children’s cheating, lying behaviors, and semantic leakage control. For example, previous research showed that different facets of ToM development predicted their respective usage in preschool years. More specifically, rudimentary forms of ToM predicted cheating behavior (e.g., knowledge access) [20,21], while lie-telling and semantic leakage control were predicted by more advanced forms of ToM, such as first-order false belief understanding for lie-telling and second-order false belief understanding for semantic leakage control [7]. Additionally, O’Connor and Evans [22] showed that preschoolers who scored higher on ToM tasks were less likely to cheat during a guessing game. At the same time, a growing body of evidence supports a positive relation between children’s propensity and proficiency to lie in such games and their performance on first- and second-order ToM tasks [23]. Such findings could be explained by the perspective-shifting that ToM allows children to make. Using various versions of the TRP task, researchers have shown that concurrently with the development of first-order ToM, preschoolers’ lies are better constructed, as they begin to understand that beliefs can be incorrect and that they have the power to instill false beliefs in others [10]. Higher ToM could predict a reduction of transgressions because children become more aware that they may get caught. Nonetheless, if the transgression does occur, children’s superior ToM skills can assist them in elaborating other lies to conceal this act (i.e., semantic leakage control). Research to date shows that semantic leakage control is related to second-order ToM [2,24], which allows elementary school children to recursively think about beliefs [25] and to progressively reason about complex relations between mental states. Based on second-order ToM inferences, children begin to carefully consider the concomitant expected values of truth and deceptive response options in a quasi-rational fashion, and thus, decide which kind of information to provide depending on the given circumstances [10].

Despite the breadth of research examining the relation between children’s deceptive abilities and first-and second-order ToM, less is known about what happens when higher-order ToM developments occur. Even less is known about how more advanced ToM relates to children’s transgressions. One of the most important post-preschool ToM developments is their understanding of interpretive diversity [4,26,27]. Lalonde and Chandler [28] defined the understanding of interpretive diversity as the ability to understand that a perceptively ambiguous stimulus can be interpreted differently by multiple individuals, naming it interpretive ToM (ToMi), and developing a new task, the Droodle task, to measure it. The task involves the usage of ambiguous drawings which are showed to the children, asking them to decide what two naïve observers will think the drawings represent. On the other hand, Schwanenflugel and collab. [29] proposed a closely related ability termed constructivist ToM (ToMc) as an understanding that “knowledge can be more or less certain, that feelings of uncertainty are important in evaluating information, that things can have multiple meanings” (p. 288). They developed The Constructivist Theory of Mind Interview to assess this ability through a number of scenarios depicting how our cognitive processes can change the way in which different persons perceive the same situation [10]. In this study, we will use both tasks and refer to interpretive diversity understanding (IDU) as a more general ability that incorporates ToMi and ToMc.

To our knowledge, the literature linking IDU to children’s deceptive abilities is almost non-existent. Only one theoretical contribution [20] suggested that higher ToM developments, such as ToMc, could be associated with children’s dishonest behavior. When *deciding* if they should lie or not, ToMc might assist children in mentally projecting the contents and the best target of deception (e.g., “I can tell my new classmate that I was sick but not to my teacher because she will ask my mom about this, who knows I’m lying”). Anticipating multiple possibilities for various individuals can allow them to make better-informed decisions about lying or not. In addition, when *constructing* a lie, ToMc could support children’s reasoning about how their deceptive statements will be processed and received by the recipient (e.g., “How will this person react if I say that I know the correct answer to the hard question from TV?”). This is in line with Walczyk’s and Fargerson’s [10] prediction that children discover more effective ways to reduce the cognitive load associated with deception across development. ToMc also allows the understanding of how a piece of information can be interpreted differently by multiple people with different mental states but yet accepted by all (e.g., “Both my colleague and the teacher would believe that I was skipping school because I was practicing for an important contest”). When it comes to semantic leakage control, ToMc could help the child flexibly adjust a lie’s content to make it credible for different recipients. However, none of these relations were tested before in a comprehensive empirical study, and there is no information on how IDU could assist children’s cheating strategies.

### 1.2. Children’s Dishonesty, Parental, and Contextual Factors

#### 1.2.1. Parental Rearing Practices

Parental rearing practices are linked to significant milestones in child development by defining many of their interactions with the environment [30]. Unfortunately, existing research linking children’s cheating and lying to parenting behaviors yielded inconsistent results. The scarce research focusing on cheating behavior in older samples shows that college students were more likely to cheat when they perceived their mother as less affectionate and nonequalitarian [31]. The authors posited that this might be because they were less likely to develop socially acceptable behavioral alternatives throughout childhood due to this aversive socialization environment. Moreover, in academic contexts, past research demonstrated that students who experienced harsh parental disciplinary practices engaged in higher levels of academic dishonesty, such as cheating [32]. Instead, more recent research on preschoolers found no association between children’s cheating and parental behaviors [33]. However, the contrasting results may be due to the differences in measuring parental rearing behaviors; while Kelly and Worell [31] reported students’ perception of parental behaviors, Kotaman [33] evaluated parents’ reports upon their childrearing behaviors.

Parents are an essential agent in children’s developmental trajectories of lie-telling through their nurturing and socializing behaviors [34]. According to the domain of socialization framework proposed by Grusec and Davidov [35], socialization takes place across several domains and includes approaches such as guided learning, group participation, control, protection, and reciprocity, through which parents are contributing to their children’s socialization of honesty and to the development of socially accepted behaviors [34,36,37]. Guided learning and group participation can contribute to children’s ability to differentiate between truth and lies and choose accordingly. Instead, control is the most intensively studied parental aspect in relation to children’s dishonesty, suggesting a strong positive association between controlling parental practices and actual lie-telling for self-serving purposes [37,38,39,40]. In support of this theoretical framework, a recent review of 13 studies argues that lying was associated with parent-child relationships characterized by low warmth and lack of communication [41]. In addition, Baudat and collab. [42] found that parental support for autonomy was related to lower lying. Similarly, Stouthamer-Loeber and Loeber [43] found that a low level of parental supervision and discipline was related to higher levels of deception. These results are consistent with Cumsille and collab [44] findings on the lack of warmth in parent-child relationships and lying behavior.

#### 1.2.2. Socioeconomic Status

Lower socioeconomic status (SES) is thought to have numerous detrimental effects, affecting children’s cognitive and language development, social functioning, and mental health [45]. Specific research on the association between SES and deceptive behavior yielded mixed results [3]. On the one hand, several studies indicated that lower SES predicts increased deception in children [46,47]. On the other hand, other research found no difference in children’s lie-telling behavior between lower and higher socioeconomic groups [43]. However, to date, no research has focused on the deception sophistication in relation to children’s SES, despite the implications for their ability to successfully deceive.

#### 1.2.3. Bilingualism

Being broadly regarded as one’s ability to use two languages in everyday contexts [48], it is difficult to provide a definitive definition of bilingualism and second-language acquisition [49]. Given the current migration patterns and socioeconomic changes around the world, it has become more common for children to learn a second language from a young age. Thus, both research and educational policy makers are interested in how this process impacts children’s socio-cognitive development [50,51]. One way of accommodating a second language is through bilingual education [52]. For instance, immersive bilingual education implies that children speaking one language at home learn their school subjects in a second language [52]. Throughout this paper, we will refer to this type of bilingual acquisition experience since our bilingual participants were enrolled in classes taught in a different language than the national language.

Undoubtedly, bilingualism influences many aspects of children’s lives. Still, the debate regarding a definitive ‘bilingual advantage’ in cognitive domains such as theory of mind or executive functioning is ongoing [53,54,55]. The relation between ToM and bilingualism represents an important research topic of the last decades, focused on what could enable bilinguals to outperform their monolingual counterparts on ToM tasks. Goetz [56] suggested that better developed executive functions and metalinguistic abilities, as well as an increased understanding of the linguistic needs of their conversation partner could enable bilinguals to better solve these tasks. With respect to lie-telling, the pioneering research investigating the relationship between bilingualism and deception is scarce and exclusively focused on the adult population, showing that using a second language can decrease the ability to accurately differentiate between truthful and deceitful statements [57]. On the one hand, this could be explained by the fact that, speaking in a foreign language regardless of the truthfulness of the conveyed message, requires more cognitive resources, which can be observed in comparable response times in both truthful and deceitful statements [57]. On the other hand, the ‘emotional distance’ hypothesis argues that people can find it easier to lie in another language, since they can to some extent separate from the emotional valence of the message [58]. To our knowledge, no study with children has addressed how bilingual children perform in peeking and lying tasks compared to monolinguals. We could anticipate a better performance considering their better ToM [59] and executive functions [60], which were documented to positively support lie-telling behavior and its complexity.

### 1.3. Relations between the Variables

In light of the theoretical framework proposed by Talwar and Crossman [3,18] regarding the importance of contextual and cognitive factors in children’s dishonest behaviors, in the current investigation we decided to zoom in on certain cognitive and contextual factors and discuss their interplay during middle childhood.

It is well established that school-age children’s increasing ability to deceive is sustained by their superior cognitive functioning [3]. Past research has widely investigated processes such as ToM or executive functioning as being related to different levels of sophistication in children’s lies [15]. In particular, advanced forms of ToM, such as IDU, are believed to be involved in every step of producing a lie (e.g., decision, activation, construction, action) [10,19]. However, there is no empirical evidence on how IDU assists children in their deceptive behavior, from less sophisticated acts, such as cheating, to more complex ones, such as semantic leakage control.

In spite of the influence of cognition upon deception, it is also well established that children’s social experiences and environmental factors can affect their honesty-related behavior, too [61]. Therefore, factors such as parental practices or socioeconomic status (SES) were also investigated in relation to children’s deception. For instance, previous research demonstrated that adolescents who perceive their parents as controlling may use deception to gain autonomy [40] or to deal with unfair restrictions on personal activities imposed by parents [62]. Additionally, adolescents who perceive their parents as controlling may be less likely to internalize the value of honesty [40].

With regard to SES influence on children’s deception, results are mixed and mainly focused on the reported frequency of children’s acts of dishonesty [43,46]. One possible explanation could be the indirect effect of SES on other predictors of children’s social development, such as parental practices. Looking at the relation between SES and parental practices, Hoff et al. [63] concluded that some aspects of parenting appear to be more susceptible to the influence of SES than others. A significant component of the SES-related differences in parenting can be attributed to parents’ styles of verbal interaction. In comparison, SES-related differences in nonverbal interaction are fewer. For example, a pervasive difference is the tendency of lower-SES parents to be more controlling and punitive than higher-SES parents [63].

Further evidence suggests that children’s advanced ToM developments (i.e., IDU) are less susceptible to the influence of parental practices and SES. For example, O’Reilly and Peterson [64] showed that school-age children’s first- and second-order false belief understanding were insignificantly associated with usual parental measures (e.g., control, rejection, warmth). Additionally, Tafreshi and Racine [65] reported the lack of association between children’s interpretive ToM (ToMi) and parental reports of permissiveness or authoritativeness. Likewise, very small associations were also reported in a recent study regarding parental warmth and rejection in relation to ToMi and ToMc [66]. Concluding on this matter, Foley and Hughes [67] posited that normative variations in parent-child relationships are not very influential for children’s development of advanced ToM. Instead, significant differences are present in instances of parental neglect and maltreatment, which are off the normative chart. The same authors pinpoint a low to modest association between normative SES variability and individual differences in ToM during school age. In turn, we have very recent longitudinal evidence that for children living in poverty, the development of affective ToM is more salient [68].

**Figure 1:**
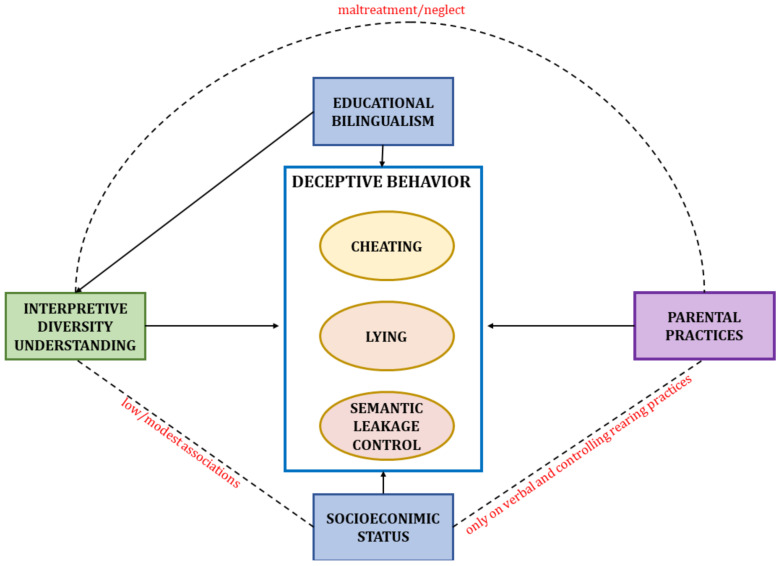
The relations between the variables of the study.

Within the European educational context and due to the current political context, which leads to the influx of migrants and new policies worldwide, another increasingly important social factor that could shape children’s ability to deceive is bilingualism. To our knowledge, the only empirical evidence on the association between deception and bilingualism comes from adult samples and suggests that bilingualism facilitates dishonesty due to lower emotional arousal when lying in a foreign language [69]. Another indirect path through which bilingualism can impact children’s deception is ToM. Past research documented higher levels of ToM performance in bilingual children than in monolingual ones [59] (see Figure 1 for the graphic representation of these relations).

### 1.4. The Current Study

We investigated the associations between various socio-cognitive, parental, and contextual factors and school-age children’s cheating, lying behavior, and semantic leakage control. First, we wanted to explore the relation between children’s cheating, lie-telling, and semantic leakage control and their interpretive diversity understanding (IDU). To the best of our knowledge, this relation has not yet been directly addressed [20]. Given this aim, we chose to study children between 9 and 11 years old because, according to previous literature on children’s understanding of mental processes, they come to understand specific mental activities gradually. For example, Lovett and Pillow [70] showed that for children is easier to understand the process of memorization before the one of comprehension, and that this understanding starts from the age of 8 and progresses intensively soon after this emergence point [71]. Consequently, we developed a new version of the TRP task to simultaneously evaluate children’s cheating, lie-telling, semantic leakage control and IDU, aiming to explore their interrelation. Although there is no previous empirical evidence on the relation between IDU and children’s deceptive behavior, based on previous theoretical arguments discussed before [20], we anticipated children’s dishonest behavior (including cheating, lie-telling, and semantic leakage control) would be positively associated with IDU.

The relation between children’s dishonesty and bilingualism was also explored. In that respect, we were interested in testing the direct and indirect effect of bilingualism on children’s dishonesty. Besides its’ direct effect, given previous literature indicating that bilingualism is associated with higher ToM performances in children [59], we anticipated a mediation effect of IDU on the relation between bilingualism and children’s dishonest behavior (cheating, lying, and semantic leakage control). 

Additionally, we hypothesized that children’s cheating and lying behavior would be negatively associated with socioeconomic status (SES) [47], so that children with higher SES will be less likely to cheat and to lie about doing so, while those with lower SES have multiple motivations for covering their misdeeds and dishonesty. Children’s cheating and lie-telling were expected to be positively associated with parental rejection and overprotective rearing practices [40,43]. We also explored the relation between children’s semantic leakage control and parental practices such as parental rejection and overprotection. Lastly, considering the previous literature demonstrating the importance of SES on certain parental practices [63] (e.g., parental verbal interactions style), we also wanted to explore the mediation effects of parental rearing practices (e.g., emotional warmth, rejection, and overprotection) on the relation between SES (income, parental education) and children’s dishonesty (cheating, lie-telling, and semantic leakage control).

## 2. Materials and Methods

The current investigation represents a cross-sectional correlational study in which we used a behavioral task in order to evaluate children’s cheating, lie-telling, and semantic leakage control.

### 2.1. Participants

Participants were recruited from different schools upon invitation to participate based on the institutional collaboration protocols and parental informed consent. We targeted schools from different urban parts of the country and selected them based on their availability and willingness to be involved in the project. From those schools, we invited all children between 9 and 11 years to participate. Consequently, only certain classes from each school were involved (e.g., classes from the 3rd and 4th grades). Informed consent was asked from children’s caregivers, but their involvement was voluntary (children and their parents were not renumerated). We received informed parental consent for a sample of 196 children, ages 9- to 11-years old (M_age_ = 124.18 months, SD = 7.25; 106 girls). In all, 113 were enrolled in monolingual schools from Northeast Romania, whereas the other 83 children attended a bilingual German- Romanian school program where they spoke German. Children’s verbal assent to participate in this study was obtained before their involvement in the testing sessions. Children who did not have written parental consent were not included in the present study.

### 2.2. Materials 

#### 2.2.1. Cheating, Lie-Telling, and Semantic Leakage Control

The Preference Task, a modified version of the Trivia Game [13], was developed to elicit children’s cheating, lie-telling behaviors, and semantic leakage control while requiring different IDU levels (low versus high). The game contained five trivia questions and was presented in an E-Prime slide show. Each slide showed a question with three possible answers. The correct answer was displayed on the following slide. Children were told that for some of the questions, they would be asked to come up with plausible explanations for the given answer to win the game and obtain a desirable prize.

The game could be played by pressing a key for going forward and another key for going backward through the slides. At first, the experimenter demonstrated this and then asked the child to navigate through the game by themselves. 

The game started with two “control” questions meant to accommodate children with the game’s rules. These were considered control questions due to their low level of complexity, simply asking children for easy answers known as common knowledge (e.g., the capital of their country). Moreover, in terms of IDU requirements, the first three questions did not elicit high IDU levels (Q1, Q2, and Q3; e.g., Q1: *Which of the following is the capital city of Romania? a. Bacau, b. Timisoara, c. Bucharest)*, while the last two required reasoning about different perspectives (Q4 and Q5; e.g., Q4: *A group of children and their parents were asked by researchers which of the following animals was the loveliest to have? a. Koala, b. Dog, c. Duck*). 

For the two questions that required high levels of IDU (Q4 and Q5), children were asked to answer by considering the perspective of two groups (children and their parents) and explaining each answer. Participants were told that, even though children and their parents had the same answer to the question, they did not always have the same reason for choosing it, thus tapping into understanding multiple perspectives of different targets. Q4 was designed as another “control” question, as it had an easy-to-know answer. However, in order to motivate their answer from two different perspectives, children had to minimally employ their interpretative reasoning when considering that parents’ responses might differ from children’s. This was meant as an IDU practicing question to prime participants on how to answer the last question, which was an “impossible to answer” question in the absence of cheating demands (Q5: *A group of children and their parents were asked by researchers about what kind of music they think is the most fascinating? with the possible answers being a. Agrotech, b. Folktronica, c. Neurofunk).*

To elicit cheating and lying, two of the questions were made up, so they were considered impossible to respond to without peeking at the correct answer because there was not a real correct answer to them (Q3 and Q5; e.g., Q3: *Who discovered Tunisia? a. Alexander the Great, b. Vasco da Gama, c. Profidius Aikman*). For these two questions, before the child answered each question, the experimenter excused themselves and left the room for 3 min, saying that they must take an important phone call, thus creating the opportunity for the child to cheat. If the child peeked by moving on to the slide in the experimenter’s absence, they would find an impossible-to-know answer on the slide. Upon return, the confederate asked the child if they peeked at the correct answer, and then the child was invited to give their answer to the respective question (i.e., to Q3 or Q5) [13].

**Figure 2:**
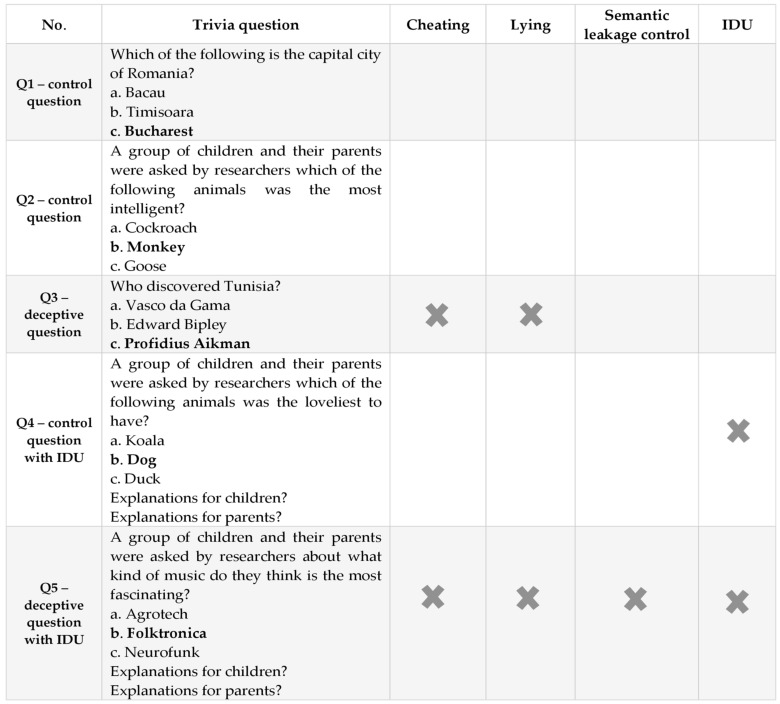
The Preference Task questions and their requirements in order to know the correct answer to each of them.

Subsequently, we had one deceptive question with low IDU level requirements (Q3) and another one eliciting high IDU levels (Q5). For Q5, if the child transgressed by moving on to the next slide in the experimenter’s absence, they would find an impossible-to-know answer on the slide along with the justifications for the children’s and their parents’ answer (e.g., *The correct answer is: b. Folktronica; Explanations: Children: Folktronica is the most fascinating because it is easy to dance to; Parents: Folktronica is the most fascinating one because it combines multiple genres*). Those who transgressed and denied their action had to generate different plausible justifications from those found in the following slide to be credible and win the game. After giving their answers, participants were shown the last slide containing the correct answer and the justifications given by children and parents (see Figure 2. for a summary of the task).

Children’s peeking behavior on the two deceptive questions was recorded by registering the keys pressed by children in the experimenter’s absence in the E-prime task. The adequacy of this new version of the task was initially piloted on an initial sample of 20 children, which led to various task refinements. Based on their behavior, children’s actions during the experimenter’s absence were scored as 2 if the child peeked on both occasions, 1 if they peeked only once, or 0 if they did not peek at all. Likewise, children’s lie-telling behavior was scored as 2 if they lied about peeking on both occasions, 1 if they lied about peeking only once, or 0 if they did not lie at all.

Also, a distinct score was obtained based on children’s given justifications for Q5 (dishonesty and IDU eliciting) and used as a proxy for semantic leakage control. We considered this score an indicator of children’s semantic leakage control because, in order to maintain the initial denial of peeking, children must be able to feign ignorance by giving different explanations than those presented to them on the slide. Children’s justifications were coded according to their match to those written on the last slide of the game (2 = entirely distinct explanations, e.g., *Children chose Folktronica because they listen to it in school. Parents chose Folktronica because it reminds them of their youth*; 1 = partially distinct justifications, e.g., *Children chose Folktronica because they often dance to it. Parents chose Folktronica because it reminds them of their youth*; 0 = identical explanations to those on the slides).

#### 2.2.2. Interpretive Diversity Understanding (IDU)

**Droodle Task**. Children’s IDU was assessed using the Droodle Task [28], which taps into children’s ability to understand that people exposed to the same stimuli can construct diverse interpretations due to their previous beliefs, attitudes, and knowledge (ToMi) [72,73]. First, children were shown a picture representing the first Droodle (e.g., an elephant and an orange) and asked to describe it. Then, the confederate fitted the drawing into an envelope into which a small viewing window was cut. This way, it masked most of the extended picture, exposing only a part of the drawing which was ambiguous (e.g., the trunk of the elephant and a part of the orange). Next, children were introduced to two dolls (i.e., naïve observers) who did not see the drawing beforehand. After that, children were asked to infer the interpretation of each doll upon the identity of the full drawing based on the ambiguous keyhole view, thus requiring them to ignore the information they had about the true identity of the drawing and to imagine two new interpretations that the dolls might have. A second trial immediately followed with a different picture.

The participants’ responses to each Droodle were coded according to the following criteria: (a) the similarity of children’s response with the original picture (1 = no similarity, 0 = obvious connection to the picture) and (b) the similarity between the attributions for the two dolls (1 = no similarity between the dolls’ descriptions, 0 = similar descriptions).

**The Constructivist Theory of Mind Interview**. Another independent measure of IDU was The Constructivist Theory of Mind Interview [71], which was meant to assess children’s capacity to reason about how a person is making sense of a situation depending on the mental processes involved and how children understand the inner workings of these processes (ToMc). The questionnaire contained 10 scenarios confronting one or two persons with visual, auditory, or verbal stimuli. Children were asked about the person(s)’ mental processes regarding those stimuli, reflecting their IDU across six different cognitive processes: Memory, Attention, Comprehension, Comparison, Planning, and Inference. Memory entails individual differences in how people remember things that happened or not (e.g., *Could two people watch the same thing happen and both see and hear everything but remember it very differently?*). Attention involves one’s ability to reflect on how people can operate with visual or auditory stimuli and make sense of them (e.g., *Can somebody look at something but not see it?*). The Comprehension scenarios question whether people can form a clear mental representation of a given material based on previous knowledge or current disposition (e.g., *Could somebody remember everything someone said to them but not understand it?*). Comparison involves contrasting different aspects of information from the world, whereas Planning involves anticipating action in relation to a predetermined goal. Finally, Inference refers to one’s ability to understand that people can come up with a conclusion regarding a situation based on different reasoning processes.

The responses were coded as “Yes, with Active mental Process Explanation” (scored as 2; e.g., *Yes, if one sees things positively, one negatively*) if children’s responses referred to the inherent differences of mental processes across individuals. However, if children made references to perceptual stimuli properties or knowledge differences between individuals, such as poor quality of perceptual information (e.g., *Yes, if one didn’t pay attention*), or if their response was Yes, but failed to explain (e.g., *Yes, but I don’t know how*), their responses were coded as “Yes, with Non-Active Mental Process Explanation” (scored as 1). Lastly, children’s lack of response or “*I don’t know*” answers were scored as 0. Six different ToMc scores corresponding to each mental process were calculated. 

#### 2.2.3. Parental Rearing Practices

Children’s perception of their parents’ behaviors was assessed using the Romanian version of EMBU – A [74], an adaptation of the EMBU [75]. The EMBU version used in the present study contained 49 items corresponding to Emotional Warmth (e.g., *Do you feel that your father/mother minds helping you if you have to do something difficult?*, Rejection (e.g., *Does your father/mother punish you for little things?),* and Overprotection (e.g., *Do you have to tell your father/mother what you’ve been doing when you get home?*) factors. The questions were answered on a 4-point Likert scale indicating the frequency to which parents were displaying those behaviors. Children first assessed the mother and then the father’s rearing behaviors with two identical questionnaires. A composite score was calculated for each EMBU factor by obtaining the average between children’s reported scores for mothers and fathers.

#### 2.2.4. Bilingualism

Immersive bilingual education was used as a proxy for bilingualism assessment. In the current study, we included children who were attending monolingual (*n* = 113) and dual-language (*n* = 83) school programs. According to this criterion and the sociodemographic information offered by parents regarding the number of languages spoken at home, we qualified participants as monolingual or bilingual. For the monolingual group, we only included children who spoke only the maternal language at home and at school, and who were not attending any intensive language courses outside of school. For the bilingual participants, we recruited children who were speaking German at school (the school subjects were taught in German), but a different language at home (e.g., Romanian, Hungarian).

#### 2.2.5. Socioeconomic Status

Besides basic sociodemographic information and languages spoken at home, parents completed a demographic survey that contained information about their education level and income. Income was assessed using a 5-point Likert scale indicating different levels of household incomes (1 = below 300 RON, 2 = between 400 and 500 RON, 3 = between 500 and 1000 RON, 4 = between 1000 and 2000 RON, and 5 = above 2000 RON). Parental education (mothers’ and fathers’) was evaluated on a 9-point nominal scale containing the formal education options available in Romania (1 = Primary School, 2 = Secondary School, 3 = Professional School, 4 = Pedagogical Highschool, 5 = Theoretical Highschool, 6 = Post-secondary School, 7 = Bachelor Degree, 8 = Master’s Degree, and 9 = Doctoral Degree). Parents had to choose one of the 9 possible options depending on the last formal education level graduated.

#### 2.2.6. Procedure

At first, we obtained parental written consent for children’s involvement in the study. Before obtaining parental consent, parents received brief information about what we were interested in investigating in the current study. They also had to complete a questionnaire regarding demographical information. Next, children with parental consent were asked for verbal assent and then completed the parental practices questionnaire in a classroom setting with the teacher’s permission.

Next, every child went through an individual testing session in which the Droodle Task, Preference Task, and Constructivist Theory of Mind Interview were administered. The whole session lasted for about 40 minutes for every child. For bilingual children, all the tasks were administered in German by a trained research assistant. As for monolingual children, the testing sessions were administered in Romanian. At the end of the session, participants went through a short debriefing session about the game, and all of them received a small reward (as promised in the deceptive game’s scenario). All the testing sessions took place in children’s schools with the teachers’ permission.

## 3. Results

First, descriptive statistics were computed (see Table 1). Second, because very few children peeked just once at the correct answers in the dishonesty task (*n* = 24), children who peeked once and those who peeked twice were collapsed in one category representing children who cheated at least once (see Table 2 for frequencies). Three binomial logistic regression were employed to test the influence of socio-cognitive factors on children’s cheating, lie-telling, and semantic leakage control. To test for all the indirect effects, we performed mediation analyses using PROCESS (model 4). Lastly, we address the possibility of multicollinearity in our data by computing bivariate correlations (see Appendix A). The correlations revealed modest associations between our independent variables which informed us that no multicollinearity was present.

A preliminary analysis explored the effects of gender. However, no main gender effects were obtained, and thus it was no longer included in the following analysis. For SES, descriptive statistics showed that 50% of the parents reported household incomes above 2000 RON. In contrast, another 25% reported revenues between 1000 and 2000 RON. This informs us that our sample comes from rather low- and middle-income families, given that the average household income in Romania is above 3500 RON [76]. In terms of parental education, data showed that approximately 40% of the parents had a Bachelor’s degree, and 12% had a Master’s Degree.

### 3.1. Children’s Peeking Behavior

Out of 196 participants, 80 (40.8%) peeked at least once at the “impossible” answers of the game. To test the effects of demographics, cognitive, parental, and contextual factors upon children’s propensity to peek, a binomial logistic regression was employed. Age, SES (income and parental education), IDU scores (ToMi and ToMc scores), bilingualism, and parental rearing practices were entered in the analysis as main effects. The overall model was significant, χ^2^ = 106.38, Nagelkerke R^2^ = .58, *p* = .000, indicating that income (*b* = 0.25, Wald = 12.54, *p* = .001, OR = 2.64), parental rejection (*b* = 0.14, Wald = 5.66, *p* = .017, OR = 1.14), and ToMc Comparison (*b* = 0.19, Wald = 10.24, *p* = .001, OR = 2.53) positively predicted children’s propensity to peek at least once.

Since the direct effect of bilingualism on children’s cheating behavior was not statistically significant, we did not perform the meditation analysis of IDU on the relation between cheating and bilingualism.

Lastly, given the significant effects of income and parental rejection on children’s peeking behavior, we employed a mediation analysis to test for the indirect effect of income on peeking behavior as a function of parental rejection. Results showed that the indirect effect of parental rejection on peeking behavior was significant (*b* = .0537, CI 95% [0.004; 0.187]), while the direct effect of income on peeking behavior remained significant (*b* = 0.943, z = 4.861, *p* = .000, CI 95% [0.563; 1.323]).

### 3.2. Children’s Lying Behavior

Among children who peeked at least once (*n* = 80), 68 (85%) of them lied about doing so. Similar to peeking behavior, because fewer children decided to lie only once (24%), children who lied once and those who lied twice were collapsed in one category representing children who lied at least once. To test the effects of demographics, cognitive, parental, and contextual factors upon children’s lie-telling behavior, a binomial logistic regression was employed. Age, SES (income and parental education), ToMc scores, bilingualism, and parental rearing practices were entered in the analysis as main effects. The overall model was significant, *χ*^2^ = 44.81, Nagelkerke R^2^ = .76, *p* = .000, indicating that maternal education (*b* = 0.347, Wald = 5.08, *p* = .023, OR = 5.11) was positively associated with children’s decision to lie. ToMc Comprehension (*b* = −0.391, Wald = 4.72, *p* = .030, OR = 0.08) and ToMc Memory (*b* = 0.520, Wald = 5.20, *p* = .023, OR = 36.84) scores were also significant predictors of this decision. The ToMc Comprehension score was a negative predictor, being related to a lower propensity for children’s lie-telling behavior, whereas the ToMc Memory score was a positive predictor. With regard to contextual factors, bilingualism (*b* = 0.429, Wald = 4.25, *p* = .039, OR = 1031.31) and parental rejection (*b* = 0.842, Wald = 3.22, *p* = .043, OR = 3.09) positively predicted participants’ decision to lie.

Given that the bilingualism effect was significant, a simple mediation analysis was performed in order to account for a possible indirect effect of IDU on the relation between bilingualism and children’s lie-telling behavior. The results showed that the indirect effect of ToM Comprehension on lie-telling was significant (*b* = .562, CI 95% [0.018; 1.550]), whereas the direct effect of bilingualism on lie-telling was insignificant (*b* = 1.276, z = 1.835, *p* = .065, CI 95% [−.086; 2.640]).

We also tested the mediation effect of parental rejection on the relation between maternal education and children’s lying behavior, but the analysis yielded insignificant results (*b* = 0.055, CI 95% [−.122; .636] for the indirect effect). The direct effect of maternal education remained significant (*b* = 0.423, z = 1.994, *p* = .046, CI 95% [.007; .839]).

### 3.3. Children’s Semantic Leakage Control

Within the sample of children who denied their transgressions (*n* = 68), a binomial logistic regression was employed to determine the predictors for children’s semantic leakage control. Because very few children partially controlled their semantic leakage control (*n* = 6), they were collapsed with children who fully controlled their semantic leakage, resulting in the category of children who controlled their semantic leakage at least once. Age, SES (income and parental education), IDU scores (ToMi and ToMc scores), bilingualism, and parental rearing practices scores were introduced as main effects and the overall model was significant χ^2^ = 32.05, Nagelkerke R^2^ = 0.52, *p* = .006. When looking at factors that significantly contributed to children’s semantic leakage control, results indicated that only the ToMc Planning score (*b* = 0.34, Wald = 6.73, *p* = 0.009, OR = 8.63) was a positive predictor.

The mediation effects of IDU and parental rearing practices on the relation between bilingualism, SES, and semantic leakage control were not tested because the binomial regression yielded insignificant associations between semantic leakage control and these two predictors.

## 4. Discussion

In the current study we examined the cognitive, parental, and contextual predictors involved in school-age children’s cheating, lie-telling behavior, and semantic leakage control. For the first time in the literature, we intersected two facets of advanced ToM (ToMi and ToMc) with children’s dishonesty by investigating them as interpretive diversity understanding (IDU). Our main findings showed that children’s decision to peek was positively related to their ability to understand the active nature of mental comparison (ToMc Comparison) and to some parental and contextual factors, such as parental rejection and income. Also, children’s decision to lie was associated with individual differences in ToMc Memory and ToMc Comprehension understanding and with contextual and parental factors such as higher maternal education, parental rejection, and bilingualism. Lastly, their subsequent ability to maintain the lie (i.e., semantic leakage control) was positively related to their capacity to understand the active nature of a decision-making process that implies planning (ToMc).

### 4.1. The Decision to Peek

Our results revealed that only 40% of the children peeked at the correct answers at least once. This represents a lower proportion than previous research reporting higher percentages (over 60%) for children’s propensity to peek [13]. However, according to Carl and Bussey [77], a smaller number of transgressions might have resulted in our scenario due to the specific nature of the deceptive task. Specifically, the authors anticipated a change in children’s behavior based on fundamental differences in the deceptive context created (cheating on a game versus cheating on a test), with fewer children cheating when the task was presented as a knowledge test rather than a guessing game. Our task was advertised as a game, but its design resembled a knowledge test. Moreover, the testing session took place in the participants’ schools. Hence, children may have perceived it as a more formal activity. As such, peeking in this context might have been regarded as frowned upon, given the moral standards imposed by such institution, resulting in fewer peekers. In order to better understand the influence that the testing environment has on children’s cheating, future research may compare children’s cheating behavior tested in schools to others tested in a more neutral setting.

In accordance with the current study’s first hypothesis, we showed that children’s cheating behavior was significantly associated with their IDU performance. More specifically, our findings suggest that children who decided to peek had higher ToMc Comparison scores. According to Schwanenflugel and colleagues [78], the mental activity of comparison involves contrasting different aspects of information from the sensory world and interpreting things differently based on one’s knowledge and experience. Perhaps understanding that people’s perceptions of the same thing can differ depending on their capacity to sample and contrast information made children more prone to peek at the correct answers. More specifically, this ability could assist them in anticipating that the experimenter could make sense of the peeking context via careful consideration of alternatives, comparing the information provided on the slides to those that a school-age child may possess. Thus, maybe children with better ToMc Comparison understanding predicted that the experimenter wouldn’t find their knowledge suspicious and would assess their knowledge as possible compared to other children.

The intriguing positive association between SES and children’s cheating was contrasting our initial hypothesis regarding the association between these two, but echoed past research. For example, Alan and collab. [79] demonstrated that children from higher SES families cheated more in a creative task than those from lower SES families. In the current study, we must consider the effects of higher income on various aspects of children’s lives. For instance, children from low-income families have less access to a computer at home. In addition, data show that even if they can afford a computer, low-income children tend to use it less than others [80]. This is a crucial aspect due to the computerized nature of our deception game. Considering this, perhaps children from lower SES families were less familiar with computer use, which, in turn, could impact their performance in the game we played, making them more reluctant to manipulate the keys meant to be pressed in order to find out the correct answers to the impossible questions. Moreover, a growing body of evidence suggests that SES can have an important impact on specific parental practices [81,82]. Compared to higher SES families, parenting within low SES families has been documented to be harsher and more controlling across cultures [83]. In the Romanian population, it was shown that parents from low-income families impose harsher discipline and controlling behaviors upon their children than those from middle-income families [84,85]. This is also sustained by our results regarding the mediation effect of parental rejection on the relation between income level and children’s cheating behavior. In the present context, it is possible that children with higher SES were more prone to peeking based on their willingness to break the game’s rules, anticipating less punishment from their parents regarding the transgression [84]. 

Lastly, we also anticipated that children’s cheating would be positively associated with parental rejection and overprotection. Our findings showed that children’s reported parental rejection scores were positive predictors of their peeking behavior, which are supported by previous research showing that children’s perceived levels of parental rejection represent a significant predictor of their externalizing behaviors [86,87]. With respect to cheating, previous research regarding the relation between students’ perceptions of parental behaviors and cheating showed a positive association [31]. In this case, children’s likelihood to peek could be facilitated by parental rejection, this kind of behavior being regarded as a way to escape parental influence or defy authority, which could also have an impact on other forms of dishonesty, such as academic cheating [32]. Moreover, this could also be explained by the studies showing that such parental behaviors are associated with cognitive deficits, such as poorer executive functions which could account for children’s peeking behavior (i.e., lower levels of inhibition) [17]. 

### 4.2. The Decision to Lie

Following the first aim of the present study, we analyzed the association between children’s lying behavior and interpretive diversity understanding (IDU). Our results showed that the ToMc Comprehension score negatively predicted children’s lie-telling behavior, whereas the ToMc Memory score was a positive predictor of this decision. Previous research argues that children’s ability to distinguish between the cognitive processes of memorization and comprehension develops gradually, studies indicating a rudimentary differentiation between them which starts to emerge even from the age of 8 and intensively progress soon after that [70,71].

Past research shows that the criteria for achieving comprehension can be both psychological and behavioral. For example, if a person has to assemble a piece of furniture, the psychological marker of comprehension would be the sense of a clear and consistent representation of the meaning of the assembling instructions. As for the behavioral markers, that would be the execution of the instructions read on the paper [70]. While its psychological features consist of having a clear mental representation or understanding the meaning of a particular situation, stimulus, or text, it was demonstrated that it is harder to define its behavioral markers, depending on the context of the activity [70]. When referring to how children come to understand the mental process of comprehension, research showed that they are more likely to emphasize the external cues that can mediate it [78]. Transferring this reasoning to the deceptive context created in the current study, we can speculate that children made sense of the experimenter’s comprehension process regarding their transgression based on the external contextual cues available. For instance, those children who identified the role of external cues in the experimenter’s comprehension process subsequently decided not to lie (e.g., *The experimenter could easily find out if I peeked or not if they can check which keys I pressed while they were away*). 

Regarding ToMc Memory, current findings showed that children’s ability to consider the constructive nature of someone’s remembering process positively predicts their lying behavior. In the present context, understanding that remembering (as depicted in the ToMc Interview’s scenarios) is subjective, dependent on one’s experience and interpretations could stimulate children’s decision to deceive. From this point of view, understanding that memory is constructive or different across people can assist children in imagining that the experimenter could consider their ability to remember such difficult facts as varying from one child to the other, thus they wouldn’t find their better performance suspicious. Moreover, IDU could assist children in mentally projecting multiple possibilities and contents depending on their assessment of how information could be remembered and considered by the recipient and made them feel more confident in their ability to lie to win the prize [29,88].

Our investigation also revealed that bilingual children were more likely to lie than monolinguals. Based on existing research, we can speculate several explanations for this finding. One promising perspective regards the “metalinguistic awareness”, which refers to bilingual children’s grasp of the fact that words are instrumental and their mental representation can vary from one person to the other (i.e., one object can have multiple linguistic labels). This ability is considered an underlying mechanism of an enhanced ToM, but more evidence is needed to directly support this claim [54,55]. In the current study we provided preliminary evidence that would sustain this perspective, as ToMc Comprehension mediated the relation between bilingualism and children’s lie-telling. Another perspective regards the “socio-pragmatic” aspect of bilingualism. Bilingual children learn from a very young age that not every person can speak the same language(s) as them; hence, they need to adapt their language to the other person’s communicational needs. As such, both metalinguistic and socio-pragmatic accounts could contribute to a more nuanced ability to understand that people can hold different mental representations [53,55]. Based on these findings, we suggest that bilinguals might be able to use their interpretive skills more easily than their monolingual counterparts within social interactions that might involve deception.

Another hypothesis of the current study was that lie-telling behavior would be negatively associated with SES. Although other studies examining the relation between SES and lie-telling behavior showed that children with lower SES lied more frequently [46], the current study revealed a positive relation between these two, as children with highly educated mothers were more likely to lie. We also tested for the association between children’s lie-telling and the other SES proxies included (e.g., income), but the results were not significant. Our significant finding regarding maternal education is consistent with other evidence suggesting that highly educated mothers tend to show more support and encourage children’s autonomy, with less harsh and controlling rearing tendencies that would guide their actions [63]. Therefore, children with highly educated mothers could feel more confident in their right to obtain the desirable prize, knowing that they are granted more freedom and understanding from their parents. Nevertheless, our mediation analysis was insignificant, revealing no indirect effects of maternal education on children’s lie-telling as a function of parental rejection. This might be because, in this age range, the SES-related differences in parental behaviors could be more evident for the controlling practices and not for the rejective ones [63].

Lastly, in line with previous literature showing a positive relation between children’s propensity to lie and parental rejection and controlling influences [40,43], we predicted that participants who were more willing to lie would also report higher levels of parental overprotection and rejection. Our results showed that children who perceived higher levels of parental rejection decided to lie more. Also, this could be explained by previous research showing that children with dismissive mothers tended to lie more as a behavioral strategy that allowed them to avoid negative consequences associated with a transgression [43]. In time, this social strategy may become a pervasive one that children adopt when faced with an authoritative figure, trying to avert possible repercussions. 

### 4.3. The Semantic Leakage Control

With regard to children’s ability to maintain their initial denials, our study showed that children’s ToMc Planning score positively predicted their semantic leakage control. Understanding the importance of planning in the generation process of a mental interpretation could have assisted them in planning their answers in the deceptive context depending on the recipient’s perspective and interpretation of things. This allowed children to flexibly adjust their subsequently given explanations considering that others might interpret things differently. Moreover, the extensive line of research investigating the influence of executive functions upon children’s lying sophistication has shown that children’s planning abilities are helping them find the best strategies to maintain their lies, previous studies demonstrating better planning performances among lie-tellers than confessors [7,89]. 

However, there were no significant effects of parental rearing practices, bilingualism or SES on children’s semantic leakage control. One explanation for the lack of significance could reside in the importance of cognitive factors for children’s ability to tell sophisticated lies in middle childhood (i.e., semantic leakage control). If peeking and telling an initial lie is decision-based, sustaining the initial lie is less a matter of decision and more a matter of skill. According to some scholars [10,20], children’s semantic leakage control could be strongly supported by their advanced cognitive development, such as ToMc, which allows them to flexibly adjust an initial lie by considering multiple scenarios and modifications for various targets across time. We also know from previous literature that ToM development in middle childhood and adolescence is not that susceptible to parental influences [64,65] or normative SES variations [67]. These theoretical arguments are supported by present findings demonstrating that sematic leakage control was positively predicted only by ToMc processes (planning).

### 4.4. Limitations

Despite the notable findings of this research, we should also pinpoint its significant limitations. Our results showed that the proportion of peekers and non-peekers was approximatively equal (40% of children peeked at least once) [77]. In spite of this high variability, we obtained significant results concerning the association between cheating and socio-cognitive factors in middle childhood. Nevertheless, in the subsequent analyses performed for children’s lie-telling and semantic leakage control the data variability of the outcome was much lower (85%, and 60% of participants, respectively engaged in lie-telling and demonstrated semantic leakage control), which affected the possibility to highlight the predictive value of the socio-cognitive and contextual factors [77]. Second, the cross-sectional nature of the study does not capture the maturational effects in their IDU, with longitudinal studies such as Talwar and collab. [12] being optimal for describing the dynamics between the socio-cognitive variables underpinning deceptive behavior.

We introduced a new version of the Trivia peeking game that more closely resembles actual testing scenarios by relying on a novel and less invasive method of recording cheating behavior. Despite the advantages of this experimental variation (ecological validity, no need to video record children as they cheat), it can induce supplemental individual confounds such as familiarity with computer use and test anxiety. Convergent validation of this novel procedure with the classical TRP paradigm is a fruitful future direction that should be pursued to ensure its validity.

The convenience sampling procedure that we used could also be an important limitation of the present study. Requiring written parental consent for the participants’ involvement in the study, we could not ensure that every 9 to 11 years old child had the same chances of being a part of the study. We tried to amend this issue and increase the generalizability of our findings to the targeted population by recruiting children from different urban parts of the country. Our sample was further limited by including children from relatively low-income families, even if parental educational levels were somewhat high. This could be explained by the country’s socio-economic context, which does not always provide the opportunities for well-educated individuals to align their income with the educational level [90].

Finally, we acknowledge that the current study mainly addressed the direct effects of the socio-cognitive variables concerning children’s dishonest behaviors (cheating, lie-telling, and semantic leakage control). This was due to the limited previous evidence on the indirect interrelations between these variables in middle childhood. More research is needed in order to capture the true complexity of the complex network of factors influencing dishonesty among school-age children and test the indirect effects as well.

### 4.5. Implications

The central contribution of our study represents a more nuanced perspective of children’s dishonesty during middle childhood, considering its connection to important socio-cognitive factors, such as interpretive diversity understanding (IDU). As Moldovan and collab. [20] argued, IDU might significantly influence children’s deceptive process beyond preschool years. Present results support positive associations between IDU and children’s cheating, lie-telling and semantic leakage control. This cognitive ability might allow them to recognize that multiple versions of the "truth" might exist regarding a specific situation and plan their subsequent actions accordingly. These preliminary results can be relevant to the limited research on what happens beyond preschool years when more advanced forms of ToM emerge and how these developments may contribute to children’s dishonesty.

At the same time, the current study represents an extension to the parental involvement research showing the importance of their rearing behaviors for shaping children’s dishonesty. The present findings suggest that even in middle childhood, children’s interaction with caregivers may still greatly influence the behavioral strategies they use in certain situations [86]. Moreover, the fact that parental rejection represented a positive predictor for children’s cheating and lying behavior may help parents understand that in time, their behavior towards their children might be associated with their social conduct, and that is of great importance to monitor how children perceive their relationship.

Finally, the current study provides important insights into how honesty promotion strategies could be designed and implemented. For example, considering that children’s cheating and lie-telling behavior were both positively associated with parental rejection, honesty could be indirectly reinforced by parents through their rearing practices. This is supported by previous literature showing that adolescents with supportive parents are putting more value on honesty than those with controlling parents [40]. 

Moreover, the fact that different ToMc processes support children’s deception behavior represents essential new evidence for the economic framework regarding children’s deception. We know that middle-aged children are capable of making decisions about deceiving or not based on careful consideration of the mental processes involved and the costs associated [10]. This can inform the interventions that seek to manipulate these kinds of expectations in children by reducing the perceived benefits of deception [19].

## 5. Conclusions

To summarize, the current study brings together various contextual, parental, and cognitive predictors of children’s dishonest behavior for the first time in a unitary design, providing a more nuanced understanding of these social acts during middle childhood. The present findings suggest that children’s ability to understand the constructive nature of the human mind is related to their cheating and subsequent ability to lie and maintain elaborate lies. Moreover, the current investigation provides further evidence concerning the parental influence on children’s cognitively complex dishonesty. Our findings support the idea that parental rejection may fosters dishonesty while being a mediator of the relation between SES and children’s peeking and lie-telling behavior. Lastly, we provided preliminary evidence for the differences in lie-telling between monolingual and bilingual school-aged children, opening new avenues for research into this interplay.

## Figures and Tables

**Table 1:** Descriptive statistics for parental and cognitive measures.

	Range	M	SD
Interpretive diversity Droodle task	0–2	1.60	0.68
Interpretive diversity ToMc Attention	0–6	2.21	1.59
Interpretive diversity ToMc Comparison	0–2	1.35	0.86
Interpretive diversity ToMc Comprehension	0–4	2.18	1.09
Interpretive diversity ToMc Inference	0–2	1.04	0.90
Interpretive diversity ToMc Memory	0–4	2.89	1.15
Interpretive diversity ToMc Planning	0–2	1.49	0.73
Parental Emotional Warmth	0–66	34.46	7.30
Parental Overprotection	0–37	13.09	4.69
Parental Rejection	0–38	12.23	4.58

**Table 2:** Peeking, lie-telling behavior, and semantic leakage control frequencies.

Peeking Behavior (*n* = 196)			Lie-Telling Behavior (*n* = 80)			Semantic Leakage Control—SLC (*n* = 68)		
No Peeking	Peeking Once	Peeking Twice	No Lying	Lying Once	Lying Twice	No SLC	SLC Once	SLC Twice
59.2%	12.2%	28.6%	15%	23.8%	61.3%	41.2%	8%	50%

## Data Availability

The data presented in this study are available on request from the corresponding author. The data are not publicly available due to the fact that personal information was gathered and the present study involves children.

## Appendix A

**Table A1:** Bivariate correlations between lie-telling, semantic leakage control, and socio-cognitive factors among children who peeked at least once (*n* = 80).

	1	2	3	4	5	6	7	8	9	10	11	12	13	14	15	16
1. Peeking behavior	-	**0.87 ****	−0.08	0.02	**0.40 ****	−0.15	0.01	−0.00	0.05	**0.36 ****	0.02	0.07	0.12	−0.07	**0.21 ****	0.10
2. Lying behavior		-	0.07	0.01	**0.34 ****	0.01	**−0.33 ***	−0.05	**0.45 ****	**0.25 ***	−0.14	0.09	**0.30 ***	0.06	−0.10	−0.11
3. Semantic leakage control			-	0.12	0.18	0.05	0.12	0.01	0.15	0.04	0.10	0.00	−0.21	**0.30 ****	−0.09	−0.08
4. Maternal education				-	**0.24 ***	0.09	0.03	0.12	0.15	0.11	−0.17	0.10	0.03	**0.26 ***	−0.03	−0.01
5. Income					-	0.14	0.02	0.11	0.20	−0.06	0.01	**0.34 ****	0.14	0.15	0.08	**−0.25 ***
6. ToMc Droodle						-	−0.02	0.15	0.07	−0.02	**0.25 ***	0.13	0.19	**0.23 ***	−0.19	0.04
7. ToMc Comprehension							-	**0.38 ****	−0.05	−0.18	0.20	−0.09	**−0.29 ***	−0.05	0.17	0.16
8. ToMc Attention								-	**0.23 ****	0.01	0.13	0.23 *	−0.14	0.10	0.09	0.01
9. ToMc Memory									-	0.08	0.05	0.07	0.02	−0.04	−0.16	0.07
10. ToMc Comparison										-	−0.04	0.06	0.08	−0.03	−0.01	−0.09
11. ToMc Planning											-	0.17	0.05	−0.00	−0.16	0.00
12. ToMc Inference												-	0.17	**0.22 ***	0.05	−0.07
13. Bilingualism													-	−0.00	−0.09	0.08
14. Parental Emotional Warmth														-	−0.04	0.07
15. Parental Rejection															-	0.14
16. Parental Overprotection																-

Note: ** p < .05; ** p < .01; Significant results are bolded*.
